# A Thailand case study based on quantitative assessment: does a national lead agency make a difference in pre-hospital care development in middle income countries?

**DOI:** 10.1186/s13049-014-0075-x

**Published:** 2014-12-12

**Authors:** Paibul Suriyawongpaisal, Wichai Aekplakorn, Rassamee Tansirisithikul

**Affiliations:** Department of Community Medicine, Faculty of Medicine Ramathibodi Hospital, Mahidol University, Rama VI Road, Bangkok, 10400 Thailand

**Keywords:** Pre-hospital care, Lead agency, Performance, Emergency, Thailand

## Abstract

**Background:**

Emergency Medical Institute of Thailand (EMIT) has been established as a national lead agency to improve emergency medical service systems since December 2008. However up to now, there has not been any published systematic assessment of its performance to guide further policy decisions.

**Methods:**

This study assesses the 4-year pre-hospital care coverage and performance in Thailand after EMIT establishment. The assessment makes use of 1,171,564 records from a national data set for pre-hospital care i.e., Information Technology for Emergency Medical Service System (ITEMS) in 2012.

**Results:**

Comparing with historical data, we found evidence indicating the national lead agency making differences in two basic requirements of pre-hospital care i.e., the coverage was increased by at least 1.4 times higher than the majority reported figures among 11 out of the total 13 regions of the country at baseline; and mean total response time for critical-coded patients, the longest in our study, is 1.6 times shorter than previously reported figure in 2008 (48.46 minutes). Analysis of the national data set also revealed quite substantial missing values indicating a need for further improvement. When historical data was not available, we compared our findings with international figures. Over triage rate of 28.4% for advanced life support (ALS) ambulance was found which is roughly a third of that reported in Taiwan. Almost all patients were stabilized and/or treated regardless of being transferred to hospitals in contrast to the scenarios in the U.S. systems which may probably be due to different payment mechanism. Relying on circumstantial evidences, we identified probable stagnation in pre-hospital care coverage for patients visiting emergency department after the establishment of the lead agency.

**Conclusions:**

This national data assessment shows progression in certain basic pre-hospital care requirements in Thailand. However, it needs regular systematic evaluation and there is still room for improvement of pre-hospital care systems such as increasing coverage, more equitable distribution of the coverage, faster response times, especially for patients with critical code, information system, cost-effectiveness study as well as further specific qualitative researches to guide further development of policy and intervention.

## Introduction

At the end of 20^th^ century, provision of formal pre-hospital care was estimated to cover 50-75% of people around the world [1]. Recently, evidence seems to indicate some improvement of the access according to a recent survey of 13 low- and middle-income countries which revealed most countries reported a uniform emergency access number which is considered a necessary mechanism of access to formal emergency medical services (EMS) [2,3]. Nevertheless, many severely ill and injured persons still come to hospital by less-formal means, such as commercial and private vehicles or non-motorized means, usually with considerable delays and without first aid [2].

Nielsen et al. referred to a number of reports indicating barriers to improvement and expansion of EMS coverage [3]. These were lack of integration of different services, lack of standards, and lack of leadership, which involved the different systems and institutional arrangements. If doctors were expected to lead system development of emergency services, it was found that few doctors or other more highly trained professionals are interested in devoting significant portions of their career to pre-hospital care [4].

To a large extent, some authorities pointed out that these issues could be comprehensively addressed by legislation on EMS including pre-hospital care, the lack of which was mentioned as one of the most frequent barriers to EMS development [5]. Legislation can comprise elements such as establishing a statutory lead agency. It also includes setting and promoting standards on training, communications, equipment and supplies, and financing of the system. Viewed as promising case studies, Colombia and Romania have recently enacted such EMS legislation. In both cases the legislation resulted in documented improvements in the care received by victims of injury and other medical emergencies, such as decreases in pre-hospital times. Nevertheless, it was admitted that quantitative monitoring and evaluation of the components of the EMS in both countries were still in need.

Recently, two pieces of evidence identified progress and pitfalls in the development of pre-hospital care in Thailand [6,7]. The progress included increased number of trained professionals to a total of 95,000 (physicians, nurses, paramedics) and volunteers (75% of the total); expansion of ambulance units and dispatch centers; and introduction of fee-for-service payment system for pre-hospital care. All these combined had contributed to increased access to pre-hospital care from below 100,000 cases in 2006 to almost 700,000 cases in 2008. Nonetheless, discrepancy of the access across geographical region was found to be almost 6 folds in 2008. Concerning appropriateness in dispatching ambulance services, only 10% of patients attending ED got access via pre-hospital ambulances. Furthermore, despite the fact that 64% of patients visiting ED was non-traumatic cases, less than 10% of them were transported by pre-hospital ambulances.

This report made use of electronic data set to shed light on quantitative assessment of pre-hospital care development in Thailand regarding the following key questions: 1) Did Emergency Medical Institute of Thailand (EMIT) as a national public lead agency improve coverage of pre-hospital care compared with pre-EMIT period? 2) Did EMIT improve the performance of pre-hospital care in terms of acuity of pre-hospital triage, response time and case management?

## Methods

### Setting

Thailand, a lower middle income country with per capita Gross Domestic Product (GDP) of 9,000 US dollars (USD) was populated with 68 million inhabitants (34% urban) in 2009 with an aging rate above that of the global average [8]. During 2000 to 2009, rapid motorization in Thailand was accompanied by reported increased annual death tolls from road traffic injury from around 10,200 to 13,600 cases (16.9 to 19.4 per 100,000 population) according to the police statistics [9]. In parallel, non-communicable disease (NCD) also posted a major threat to public health e.g., stroke and ischemic heart diseases were ranked among the top 3 health burdens [10]. Acute episodes of NCD combined with injuries mean increased needs for EMS in terms of quantity and complexity.

**Figure 1:**
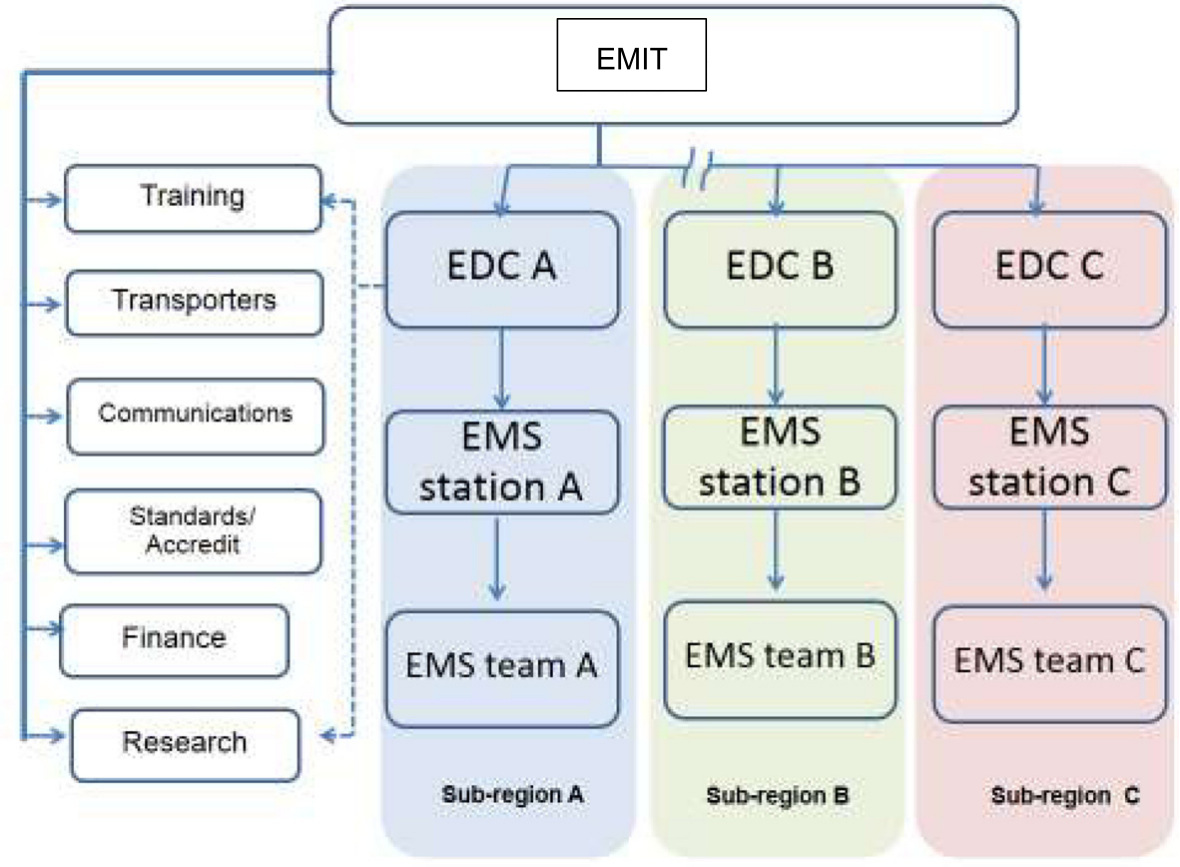
**Structures and functions of prehospital care system in Thailand.** Note: EMIT: Emergency Medical Institute of Thailand; EDC:Emergency Dispatch Center. __ Line of Command and Support. --- Line of Coordination and Information.

At the end of 2008, the Emergency Medical Institute of Thailand (EMIT) was established as a statutory national lead agency under the ministry of public health [11]. It was mandated to formulate master plans which included, to set standards of system components such as service provision, transport vehicles and personnel training curriculums; and provide financial support for service provision, research and development (Figure 1). During 2009 to 2012, the EMIT chose to focus on expansion of infrastructure and mechanisms for pre-hospital care including manpower development and setting up new ambulance stations. This was built on the momentum of earlier efforts on pre-hospital care development which started at least 2 decades prior to the establishment of EMIT. The contemporary EMS system in Thailand adopts the Anglo-American model which is based around “scoop and run” philosophy.

In the Thai publicly financed ambulance services system, the major source of finance, is formed into 4 tiers (Advanced Life Support (ALS), Intermediate Life Support (ILS), Basic Life Support (BLS), and First Responder (FR)). Each tier is staffed with different sets of staff mix guided by payment rate and standard specification defined by EMIT [12]. The operation encompassed call taking, dispatching, ambulance transport, and on-scene treatment or stabilization.

As of March 2013, there are 2,183 ALS units, 42 ILS units, 1,837 BLS units and 8,629 FR units under public hospitals, private hospitals, local authorities, and voluntary organizations [13]. During 2009–2012, the number of manpower had grown from a total of 94,634 to 122,945 of which registered FRs constituted the majority (82.7% in 2012).

### Data sources and variables

To shed light on those issues of assessment, the report made use of a data set which was Information Technology for Emergency Medical Service System (ITEMS) which operates under EMIT. The data structure comprises key variables appropriate for this study as follows, phone call numbers or other modes of call making; chief complaints such as difficult breathing, cardiac arrest, seizure; mode of transport (land, water, air); time stamps (notification receipt, dispatch, ambulance exit from station, arrival at scene, depart from scene, arrival at hospital, back to station); type of personnel delivering care; the 4 levels of pre-hospital care ambulance system; priority code (critical, moderate, mild or not emergent) assigned by Emergency Dispatch Center (EDC), using Criteria Based Dispatch protocol adapted from that of the King County, Seattle, Washington [14]; patient status on-scene was based on pre-hospital care team assessment; types of on-scene patient care (stabilization with/without hospital transfer, stabilization with death during transfer, stabilization with death on scene, operation abort, patient denial of care, missed cases, death before pre-hospital care team arrival); demographic profile of patients; and place and time of occurrence.

There are at least 2 major weaknesses of the ITEMS data set. Firstly, ITEMS did not cover the majority of operations of pre-hospital care in Bangkok, with a population of 1/6 of the total 68 million population, including those of all private hospitals throughout the country with 22% share of overall hospital beds. Secondly, ITEMS data set was not linked to inpatient care and discharge status from Emergency Departments (EDs). This precludes assessment of coverage of pre-hospital care among hospitalized emergency patients.

### Statistical analysis

There were 1,892,085 records in the ITEMS data set in 2012. After excluding records with missing values (n = 720,521 or 38%), a total of 1,171,564 records were included in the analysis. There could be a number of reasons for the substantial missing values e.g. insufficient quality control of data recording, instability of online data entry which required reliable nationwide internet network, and turnover rate of pre-hospital care staff in certain areas. The unit of analysis is pre-hospital care operation. Descriptive statistics were applied using percentage estimation or calculation of mean, median, SD, interquartile range where appropriate. All of the analyses were performed using Stata statistical software version 10 (Stata Corp, College Station TX, USA).

## Results

We found that pre-hospital care operations were 1.17 million runs (Table 1) equivalent to 17.2 runs/1000 population, with land transport mode constituted 97% of the total. ALS ambulance of land transport was assigned to 71.6% of patients coded critical and 28.4% to others with less urgent codes. This mismatch between the assignment of ALS ambulance and the patients with less urgent codes indicates over triage rate of 28.4% for the assignment. Table 2 depicts mean total response time for critical-coded patients, and the longest in our study, was 29.3 minutes (median 24 minutes). Almost all patients were stabilized and/or treated regardless of being transferred to hospitals (Table 3). Those patients with critical code were 116 times more likely to be reported dead as compared to those with not urgent code.

**Table 1:** **Percentage distribution of priority code of patients by mode of transport 2012**

**Priority code**	**Mode of transport**										
	**Land transport**				**Others**						
	**FR**	**BLS**	**ILS**	**ALS**	**AIR**	**WATER1**	**WATER2**	**WATER3**	**WATER4**	**N (row)**	**%**
critical	3.6	5.4	7.5	71.6	33.8	66.9	32.8	11.9	12.6	156,625	13.4
moderate	72.1	80	84.5	26.1	48.6	28.7	46.3	45.3	62.6	792,819	67.7
mild	20.1	12.1	6.5	1.8	12.2	3.1	7	25.2	12.1	183,322	15.6
not emergent	4.2	2.6	1.6	0.5	5.4	1.3	13.9	17.6	12.6	38,798	3.3
N (column)	752,516	219,424	37,877	159,331	74	613	540	983	206	1,171,564	100
%	64.2	18.7	3.2	13.6	0.01	0.05	0.05	0.08	0.02		

Note: water1 = motor boat with 2 engines and 2 or more FRs; water2 = motor boat with 1 engine and 2 or more FRs; water3 = long-tail boat with 1 or more FRs;water4 = other water crafts.

**Table 2:** **Response time in minutes at various steps of ambulance flows by priority code, 2012**

		**Ambulance flows**				
**Priority code**	**Duration (minute)**	**Call taking to dispatch**	**Station exit**	**Station exit to scene**	**Scene to hospital**	**Total**
Critical	mean	1.86	1.49	9.33	11.28	29.34
(N = 156,625)	median	0	1	7	9	24
	SD	27.43	8.03	10.90	16.55	34.14
	IQR	0-1	1.0-1.3	4-12	5-15	16.00-35.00
Moderate	mean	1.40	1.12	6.36	13.72	25.71
(N = 792,819)	median	0.15	1	4.51	12	23
	SD	25.53	6.66	8.69	12.56	25.41
	IQR	0-1	0.2-1.0	2-8	6-19	15.67-32.00
Mild/not urgent	mean	1.26	1.08	5.65	13.85	25.12
(N = 222,120)	median	0	1	4	12	22
	SD	22.65	6.03	7.84	12.43	23.73
	IQR	0-1	0-1	2-7	6-19	15.50-31.00

**Table 3:** **Types of prehospital care responses and patient outcomes by priority code (%) 2012**

**Priority code**	**Stabilized/ transferred**	**Treated without transfer**	**Dead during transport**	**Dead on scene**	**Dead before team arrival**	**Patient denial**	**OR for being fatal**
Critical	145,203 (12.9)	1,388 (18.4)	845 (94.1)	460 (89.5)	4,759 (87.5)	533 (5.4)	116.51
Moderate	765,035 (68.1)	4,212 (55.8)	45 (5.0)	46 (8.9)	582 (10.7)	6,819 (69.7)	2.55
Mild	176,207 (15.7)	1,600 (21.2)	5 (0.6)	8 (1.6)	90 (1.7)	2,173 (22.2)	1.68
not urgent	37,637 (3.3)	349 (4.6)	3 (0.3)	0 (0.0)	10 (0.2)	264 (2.7)	1
Total	1,124,082 (100)	7,549 (100)	898 (100)	514 (100)	5,441 (100)	9,789 (100)	

Note: OR = Odds ratios.

## Discussion

Using nationwide administrative data, the present report provides quantitative assessment of pre-hospital care performance in selected dimensions important for further development of the pre-hospital care system in Thailand.

### Expansion of pre-hospital care operation

From 2010–2012, the number of pre-hospital care operations increased from 1,213,045 runs (18.3 runs/1000 population) to 1,261,055 runs (18.8 runs/1000 population) per year [6] which are rather low as compared to internationally reported figures which varied from 0.33 to 72 runs/1,000 population per year [15]. It is likely that this increased coverage is a result of increased human resources and pre-hospital care operation units. However, comparing to reported figures in 2007 [6], the estimated figure in our study of 17.2 runs/1000 population is at least 1.4 times higher than the majority reported figures among 11 out of the total 13 regions of the country and 0.74 times that of the highest reported figure. This increased coverage is likely due to the increased human resources and pre-hospital care operation units mentioned above.

### Timeliness of pre-hospital care

Given the time sensitive nature of emergency care, response times have been commonly used to indicate performance of pre-hospital care [15]. The mean total response time for critical-coded patients (Table 1), the longest in our study, was 1.6 times shorter than the previously reported figure in 2008 (48.46 minutes) [7]. Again this improvement is in keeping with the increase in human resources and operation units. Compared with other countries, our reported median response times are in between those figures reported from South Africa (15 minutes in urban systems and 40 minutes or longer in some rural systems) [16]. However, they are longer than those for ALS ambulance reported from Taiwan (4.1-4.9 minutes in urban Taipei and up to 6.6 minutes in rural settings) [17]. It should be noted that the comparisons are not straightforward due to differences in reporting. Given the benchmark response time of 8 minutes for calls coded critical in Thailand [13], it is clear that there is plenty of room for improvement of pre-hospital care response in this regard. For instance, response times to calls with critical code could be much improved at two steps i.e., station exit to scene (7 minutes) and scene to hospital (9 minutes) (Table 2). The figures shown in our study are probably rare in international literatures, given the scope of the data covering the whole country and details of the figures in terms of priority code and steps of ambulance flows.

### Acuity of triage in dispatching pre-hospital care ambulance

To maximize resource usage, matching call priority to level of ambulance services is a crucial step. Our study revealed evidence indicating relatively appropriate matches as shown in Table 1. Comparing the over triage rate of 28.4% in our report with that of Taiwan (72.8%) [17], it was clear that our figure was much lower. This relatively high acuity is in keeping with the likelihood of being reported dead for cases with critical code as compared to those with less urgent codes (Table 3). The discrepancy between our figure and the Taiwanese figure might be a result of availability of a standard dispatcher protocol and decision making steps for delivering ambulance services. According to the Taiwanese report, these factors were not well established in many pre-hospital care systems of the country which is less likely to be the case in the Thai pre-hospital care systems described above.

In the U.S., Medicare and other payers do not pay for out-of-hospital care including response, triage, and patient assessment and treatment unless the patient is transported to an emergency department (ED) [18]. With most private insurers imitating Medicare, this payment policy significantly affects the behavior of EMS agencies contributing to an inefficient use of out-of-hospital care resources [19]. Our findings in Table 3 show a contrasting scenario in which patients at scene could be stabilized and transferred or treated without transfer due to the payment mechanism by EMIT covering staff fee, opportunity loss due to patient denial or not encountering the patient, fuel cost, medical supplies, and depreciation of investment in office and motor vehicle.

### How does pre-hospital care meet the needs of patients visiting ED?

Before the full operation of EMIT in late 2009, coverage of pre-hospital care in Thailand was estimated at 7.3 percent of visitors to ED in early 2009 based on a survey of 12 public regional hospitals [20]. In 2013, a negative correlation (−0.4 with p-value <0.001) was reported between the number of ED visits per 100 population and the ratio of the number of pre-hospital care services to that of ED visits using the ITEMS data set and emergency room data set (Sornsrivichai V: A report on gap analysis of the coverage of pre-hospital care services across provinces, unpublished). Considering these circumstantial evidences, it could be argued that so far there has not been clear evidence to support significant advancement of pre-hospital care coverage for patients visiting ED after the presence of EMIT. This is in keeping with the fact that there has not been formal linkage between ITEMS data set and ED data set rendering difficulty in getting appropriate feedback for the improvement. In effect, there has not been an exclusive ED data set in our country.

### Gaps for further improvement

Given the improvement in certain aspects of pre-hospital care as discussed, there is still plenty of room for improvement of pre-hospital care such as increasing coverage (currently 17.2 runs per 1000 per year), more equitable distribution of the coverage (a provincial disparity of almost 10 times in 2013 which is worse than the baseline figure of 7 times in 2007) [6,20] reducing response times especially to calls with critical code (17% of the 8-minute target for ALS), improving ITEMS data set by including pre-hospital care operations in Bangkok, the most populated province, and by establishing a linkage with future establishment of ED data set. Finally, there is still a need for more investment in further research such as cost-effectiveness study, knowledge translation and specific qualitative researches to guide further development of policy and intervention.

## Conclusions

Based on the analysis of administrative data of over a million records, the present study provides quantitative assessment of pre-hospital care performance at national scale in Thailand using selected important indicators. The establishment of EMIT in late 2008 as the statutory national lead agency seemed to make some differences in terms of expanding coverage of pre-hospital care and shortening response times as compared to the baseline figures. These improvements correspond with increased human resources and pre-hospital care operation units. Nevertheless, this report could not assess the effect of EMIT on other dimensions of the performance of pre-hospital care due to the lack of pre-EMIT evidence in terms of acuity of triage to dispatch pre-hospital ambulances and case management. Given the circumstantial evidence during the pre-EMIT period, this report shed some light on probable regression of equitable access across provinces and coverage for patients visiting ED who might need ambulance transport. These seemingly deficit areas need sufficient attention of EMIT. Finally, EMIT should pay more attention to further improvement of pre-hospital care such as increasing coverage, more equitable distribution of the coverage, improvement of response times especially for patients with critical code, information system, and research to guide further development of policy and interventions.
